# The Role of Avapritinib for the Treatment of Systemic Mastocytosis

**DOI:** 10.7759/cureus.18385

**Published:** 2021-09-29

**Authors:** Vikram Sumbly, Ian Landry, Saba Iqbal, Zamaraq Bhatti, Mohsen S Alshamam, Salman Ashfaq, Vincent Rizzo

**Affiliations:** 1 Internal Medicine, Icahn School of Medicine at Mount Sinai, New York City Health and Hospitals, Queens, USA; 2 Medicine, Icahn School of Medicine at Mount Sinai, New York City Health and Hospitals, Queens, USA; 3 Medicine, Allama Iqbal Medical College, Lahore, PAK

**Keywords:** tyrosine kinase inhibitor, hematology, ayvakit, avapritinib, systemic mastocytosis

## Abstract

Systemic mastocytosis is a rare hematologic disorder characterized by the clonal proliferation of mast cells in extra-cutaneous organs. This disease can be further subdivided into five different phenotypes: indolent systemic mastocytosis (ISM), smoldering systemic mastocytosis (SSM), aggressive systemic mastocytosis (ASM), systemic mastocytosis with an associated hematological neoplasm (SM-AHN) and mast cell leukemia (MCL). The tyrosine kinase inhibitor (and also potent KIT D816V inhibitor) avapritinib, initially approved for the treatment of gastrointestinal stromal tumors (GISTs) bearing a PDGFRA exon 18 mutation, also showed great promise in patients with systemic mastocytosis, a disease known to be driven by a mutation in KIT (D816V). We present an overview of this rare disorder, including a review of the current understanding of the genetic mechanisms which lead to the disease state, the action of the tyrosine kinase inhibitors, as well as the latest clinical trial data which led to the current recommendations for the use of avapritinib.

## Introduction and background

Mastocytosis is a rare hematological disorder defined by the abnormal accumulation of neoplastic mast cells (MCs) within one or more different types of tissue [1]. Mastocytosis was once considered to be a type of myeloproliferative neoplasm, but in 2016 the World Health Organization (WHO) reclassified it as a type of myeloid neoplasm. This disease can be broadly separated into three categories: cutaneous mastocytosis, systemic mastocytosis and mast cell sarcoma [2].

Systemic mastocytosis (SM) is characterized by the clonal proliferation of mast cells in extra-cutaneous organs. A population-based study suggested that the incidence of SM in the United States of America (USA) is approximately 4.6 per 1,000,000 among the general population, with the majority of patients being Caucasian and approximately 55 years old with no gender predilection [3]. This disease can further be subdivided into five different phenotypes: indolent systemic mastocytosis (ISM), smoldering systemic mastocytosis (SSM), aggressive systemic mastocytosis (ASM), systemic mastocytosis with an associated hematological neoplasm (SM-AHN) and mast cell leukemia (MCL) [4].

The clinical signs and symptoms of SM are dictated by (1) the uncontrolled release of lipid mediators, cytokines, and vasoactive amines from MCs, and (2) SM-induced organ dysfunction [5]. Most ISM patients benefit from a near-normal life expectancy and only experience mild mast cell-mediated symptoms such as flushing, pruritus, diarrhea and headaches [5,6]. In contrast, patients who are diagnosed with ASM, SM-AHN and MCL have worse survival rates due to organ damage secondary to mast cell infiltration [6]. Indeed, the advanced forms of SM have been associated with survival ranging from months to a few years [6-9].

Also known as Ayvakit, avapritinib is a small molecule kinase inhibitor approved by the Food Drug Administration (FDA) in 2021 for the treatment of advanced systemic mastocytosis (AdvSM) in adults [10]. Based on the results of clinical trials, avapritinib was initially approved for the treatment of gastrointestinal stromal tumors (GISTs) bearing a PDGFRA exon 18 mutation [11]. As a potent KIT D816V inhibitor, avapritinib also showed great promise in patients with SM, a disease shown to be driven by KIT D816V mutation (substitution mutation with aspartate to valine, position 816) [10].

## Review

Method

The first author used PubMed, Google Scholar, ScienceDirect and ClinicalTrials on August 24th 2021 to informally search for articles written in English. The following keywords and Boolean operators (“AND”, “OR”) were used for this narrative review: “Avapritinib”, “Ayvakit”, “Avapritinib OR Ayvakit AND Systemic Mastocytosis” and “Systemic Mastocytosis” (Table 1).

**Table 1:** Number of articles per search engine

Keywords or combination of keywords	PubMed	Google Scholar	ScienceDirect
Avapritinib	74	867	97
Ayvakit	75	200	7
Systemic Mastocytosis	2528	27000	6518
Avapritinib OR Ayvakit AND Systemic Mastocytosis	18	389	2

The initial search encompassed articles from 1991-2021. The chosen articles were either basic research, clinical research or translational research papers. Studies were included if they fulfilled the applied criteria: Full scientific papers written in English and published within 1991 and 2021; published in vivo or in vitro studies that discuss the genomics of systemic mastocytosis; published in vivo or in vitro studies that discuss the mechanism of action of avapritinib; published the effects of avapritinib in humans. Otherwise, studies were excluded if they met any of the following criteria: case reports or case series; editorial or opinion articles; data extracted from animals that were non-murine in origin. In total, 49 articles were selected by the first and second authors to create this review article.

Discussion

Molecular Events

The survival, development and proliferation of mast cells are dependent on the interaction between the tyrosine kinase KIT (CD117), a transmembrane cytokine receptor, and its ligand, stem cell factor (SCF) [12]. More than 80% of all SM cases are caused by the D816V gain-of-function mutation within the KIT gene. This type of mutation promotes SCF-independent KIT autophosphorylation, which in turn facilitates uncontrolled cellular growth and tumorigenesis [6]. Men were found to be more prone to the KIT D816V mutation and were more likely to acquire advanced forms of SM, translating to worse overall survival (OS) and progression-free survival (PFS) [13]. A small minority of AdvSM cases have also been associated with the V560G, D815K, D816Y, D816F, D816H, and D820G mutations in the KIT gene [14-19]. Longley et al. have suggested that KIT mutations affect the regulation of the kinase molecule and alter certain amino acids within the enzymatic domain of the tyrosine kinase [20]. Although KIT mutations are thought to be crucial in the development of SM, it is postulated that mutations in other genes, such as TET2, SRSF2, RUNX1 and ASXL1, are also required for SM to develop [13,14,21-23].

Mutations of the TET2 and SRSF2 genes predispose murine models to more aggressive forms of SM and are thought to occur before KIT D816V in SM-AHN [24-26]. TET2 mutations have been observed to occur at a frequency of 20-29% in SM [27,28]. The inactivation of TET2 accelerates the formation of hematological malignancies by enhancing hematopoietic stem cell (HPSC) survival [29]. Abnormal SRSF2 proteins also disrupt proper hematopoietic differentiation [30]. Patients with TET2 -/- myeloid malignancies have been shown to have low levels of 5-hydroxymethylcytosine as well as excessively methylated DNA [31].

AdvSM patients were more likely to harbor missense and frameshift mutations in the RUNX1 gene than ISM or SSM patients [23]. Also known as acute myeloid leukemia 1 protein (AML1), the RUNX1 gene encodes for a transcription factor that modulates the differentiation of HSPCs into mature hematopoietic cells [32]. Very little is known about how RUNX1 mutations contribute to the pathophysiology of SM, but they have been shown to induce the following biological mechanisms: genomic instability, stem cell suppression, cell cycle impairment, p53 signaling inhibition, ribosomal biogenesis suppression and oncogenic signaling pathway activation [33]. 

Mutations of the ASXL1 gene have also been reported in SM [23]. Nagase et al. have noticed that the loss of ASXL1 disrupts normal hematopoiesis and favors leukemic transformation [34]. Abnormal chromatin remodeling is thought to be an important event in the formation of leukemic cells [35]. Albeit at a lower frequency, JAK2 and RAS gene mutations were also found in AdvSM [23].

Diagnosis

Patients who are suspected to have SM should undergo a bone marrow (BM) biopsy as the presence of dense multifocal mast cells within the BM is a pathognomonic feature of SM [4]. Marrow tissue should initially be screened under a microscope for any morphological abnormalities. Neoplastic mast cells with excessive clusters of differentiation markers CD2, CD25 and CD30 are then detected via immunohistochemistry (IHC) and flow cytometry [36-38]. Other important stains are CD117 and mast cell tryptase. High sensitivity polymerase chain reaction will report any KIT D816V mutations [39]. Serum tryptase levels are also an important diagnostic tool [40]. If diagnosis of SM is confirmed, dual-energy x-ray absorptiometry (DEXA) scans, bone x-ray studies and abdominal ultrasounds may aid in staging [39].

According to the updated 2016 WHO guidelines, a diagnosis of SM requires the presence of one major and one minor criterion or three minor criteria (Table 2) [8].

**Table 2:** Major and minor criteria for systemic mastocytosis diagnosis MC: Mast cells; BM: Bone marrow

Major Criterion	Minor Criterion
1. Multifocal, dense infiltrates of MCs (≥15 mast cells in aggregates) detected in sections of BM and/or other extracutaneous organs	1. Tryptase levels > 20 ng/ml
	2. Abnormal mast cell CD25 expression
	3. Presence of KIT D816V mutation
	4. Presence >25% atypical MCs

Once the percentage of mast cells within the bone marrow has been determined, patients should then be observed for “B findings” and “C findings” as this will help in differentiating between the subtypes of SM (Table 3) [4].

**Table 3:** B and C findings in systemic mastocytosis (SM) BM: Bone marrow, ANC: Absolute neutrophil count

B findings	C findings
1. High mast cell burden shown on BM biopsy: >30% infiltration of cellularity by mast cells (focal, dense aggregates) and serum total tryptase level > 200 ng/mL	1. Bone marrow dysfunction caused by neoplastic mast cell infiltration, manifested by ≥1 cytopenia(s) (ANC <1.0 × 10^9^/L, Hgb <10 g/dL, and/or platelet count <100 × 10^9^/L).
2. High mast cell burden shown on BM biopsy: >30% infiltration of cellularity by mast cells (focal, dense aggregates) and serum total tryptase level > 200 ng/mL	2. Palpable hepatomegaly with impairment of liver function, ascites and/or portal hypertension.
3. Signs of dysplasia or myeloproliferation, in non-mast cell lineage(s), but insufficient criteria for definitive diagnosis of an associated hematological neoplasm (AHN), with normal or only slightly abnormal blood counts.	3. Skeletal involvement with large osteolytic lesions with/without pathological fractures (pathological fractures caused by osteoporosis do not qualify as a “C” finding.
4. Hepatomegaly without impairment of liver function, palpable splenomegaly without hypersplenism, and/or lymphadenopathy on palpation or imaging.	4. Palpable splenomegaly with hypersplenism.
	5. Malabsorption with weight loss due to gastrointestinal mast cell infiltrates.

If an SM patient meets the WHO criteria for associated hematological neoplasm (AHN), a diagnosis of SM-AHN can be made. If not, the total number of mast cells within the BM and the presence of B findings or C findings will dictate the final diagnosis. To diagnose mast cell leukemia, the BM will have to contain >20% mast cells [4]. The detection of at least one C finding is required for a diagnosis of ASM. ISM and SSM both lack C findings, but only SSM has two to three B findings [4]. A patient can only be diagnosed with ISM if the criteria for diagnosing SSM, ASM, MCL and SM-AHN are not met.

Avapritinib in Clinical Trials

The Federal Drug Administration approved avapritinib (Ayvakit) for advanced systemic mastocytosis in 2021. Three important clinical trials (EXPLORER, PIONEER, and PATHFINDER) have studied safety and efficacy with avapritinib use in systemic mastocytosis. The EXPLORER study is an ongoing, multicenter, phase I study in adult patients with advanced systemic mastocytosis (AdvSM) or relapsed/refractory myeloid malignancies which began in 2016. Dose escalation and expansion were evaluated in 69 patients and overall response to therapy was determined according to the International Working Group-Myeloproliferative Neoplasms Research and Treatment and European Competence Network (IWG-MRT-ECNM) protocol. Thirty-nine patients with advanced systemic mastocytosis were available for evaluation and found to have an overall response rate of 77% (30/39 patients). At 24 months, the overall survival rate was 78% in all patients with advanced systemic mastocytosis. Patients with certain mutations that are known to carry a poorer prognosis (i.e., SRSF2, ASXL1, and/or RUNX1 - the so-called S/A/R positive genotypes) also experienced strong clinical response with avapritinib with a 73% overall response rate (16/22). In patients with indolent or smoldering systemic mastocytosis (n=15), 92% had clearance of mast cell aggregates in their bone marrow and 40% of these patients had undetectable levels of mutation. These lower-risk genotypes also experienced 100% overall survival at 24 months. Avapritinib was well tolerated in this study with most adverse effects being limited to nonspecific gastrointestinal symptoms. Approximately 10% of patients studied experienced pancytopenia, which was not severe enough to force withdrawal from the trial [41]. 

The success of the EXPLORER trial led to the ongoing, randomized, double-blind, placebo-controlled phase 2 study called the PIONEER trial. The PIONEER trial sought to determine recommended doses that would be both safe and effective in patients with indolent and smoldering systemic mastocytosis, as well as to determine long-term effects and safety profiles of avapritinib. The current findings of this trial show that treatment with daily avapritinib 25-100 mg may reduce serum tryptase levels, a surrogate for B-cell activity [41]. Finally, the PATHFINDER trial, a phase 2 trial that seeks to evaluate overall response rates, survival and quality of life measures in patients with advanced systemic mastocytosis treated with daily avapritinib 200 mg, found a 75% overall response rate (interim analysis). Safety data shows that only 8% of patients discontinued the trial due to adverse effects. This study is set for completion in 2022. 

Comparatively, other tyrosine kinases such as midostaurin, dasatinib and nilotinib were associated with a 60%, 33% and 21.6% overall response rate respectively [42-44]. AdvSM patients who cannot achieve durable remissions with medical management can still benefit from allogenic stem cell transplantation (alloHSCT), but overall survival remains approximately 57% [45].

Mechanism of Action

The KIT proto-oncogene belongs to a transmembrane receptor family called receptor tyrosine kinases (RTK), specifically type-3 receptor kinases. These RTKs play a key role in cell signaling through a process known as signal transduction. Under physiologic conditions, the KIT family phosphorylates pathways which play key roles in the proliferation of multiple cell lineages. Because of their role in cell signaling, they are involved in many vital processes of blood cells, including hematopoiesis, stem cell maintenance, and mast cell development and function [46]. 

Translational research involving mutant cell lines in gastrointestinal stromal tumors (GIST) and melanoma discovered oncogenic KIT mutations which occurred in exon 11 of the KIT genome [47]. Mutations in exon 11 remove the inherent autoinhibition of the tyrosine kinase activity. This discovery led to the creation of the first-generation tyrosine kinase inhibitors (TKIs): imatinib, sunitinib, and regorafenib. First-generation TKIs bind to the inactive conformation and showed efficacy in exon 11 mutations which significantly changed the survival of GIST patients [47]. However, mutations in exons 13 and 14 (drug/ATP-binding pocket) or exon 17 (the activation loop) developed which created resistance to first-generation TKIs through a combination of three mechanisms: (1) direct steric hindrance with the TKI, (2) increased affinity of ATP for the binding site, or (3) destabilization of the conformation required for TKI binding [46]. The result of this resistance led to the discovery of the second-generation TKIs (midostaurin and avapritinib) which are capable of binding to this constitutively active conformation site. Over 90% of patients with advanced systemic mastocytosis are believed to have an acquired KIT D816V, thereby providing a potential therapeutic target [48]. 

In addition to its binding of the active conformation site, studies have found that avapritinib may restore chemosensitivity to cells that possess mutations in ATP-binding cassette (ABC) transporters ABCB1 and ABCG2. These transport proteins are known to efflux chemotherapeutic agents and studies have shown that drug-resistant cells overexpressing ABCB1 or ABCG2 were equally sensitive to avapritinib as their drug-sensitive parental cells, suggesting that avapritinib is likely a high-affinity substrate of these mutated transport proteins [49]. 

Limitation

The team did not have access to several articles which appeared to be relevant to our topic. Hence, it is entirely possible that crucial details discussed within those papers were not available for review. Furthermore, we did not discuss the various biochemical cascades involved in SM. A better understanding of these cascades may explain how various genetic mutations cause SM and lead to future targets.

## Conclusions

The purpose of this review was to review the current understanding of systemic mastocytosis and show why avapritinib has been approved by the FDA for its treatment. Systemic mastocytosis is a rare hematological malignancy that can be divided into five subgroups. Although the exact pathogenesis of this disorder is not fully understood, it has been linked to various genetic mutations. Avapritinib is a potent inhibitor of the KIT-D816V mutation and has shown great promise in different clinical trials. A larger focus on the disrupted biochemical pathways and mutated genes in murine models has the ability to elucidate pathways involved in the pathogenesis of this disease which may lead to the discovery of novel selective drugs.
